# Circular RNA hsa_circ_0000073 contributes to osteosarcoma cell proliferation, migration, invasion and methotrexate resistance by sponging miR-145-5p and miR-151-3p and upregulating NRAS

**DOI:** 10.18632/aging.103423

**Published:** 2020-07-24

**Authors:** Xia Li, Yi Liu, Xiangxin Zhang, Jun Shen, Renjie Xu, Yubo Liu, Xiao Yu

**Affiliations:** 1 Department of Orthopaedics, The Affiliated Huai’an Hospital of Xuzhou Medical University and The Second People’s Hospital of Huai’an, Huai’an 223002, People’s Republic of China; 2 Department of Orthodontics, School and Hospital of Stomatology, Cheeloo College of Medicine, Shandong University, and Shandong Key Laboratory of Oral Tissue Regeneration, and Shandong Engineering Laboratory for Dental Materials and Oral Tissue Regeneration, and Shenzhen Research Institute of Shandong University, Jinan, Shandong, People’s Republic of China; 3 Department of Orthopedics, The Affiliated Suzhou Hospital of Nanjing Medical University, Suzhou Municipal Hospital, Suzhou 215002, People’s Republic of China

**Keywords:** osteosarcoma, hsa_circ_0000073, miR-145-5p, miR-151-3p, NRAS

## Abstract

An increasing number of studies have demonstrated that circular RNAs (circRNAs), as promising therapeutic targets, are essential for diverse human diseases, especially cancer. However, the potential functions and complex mechanisms of most circRNAs in osteosarcoma (OS) are still not fully elucidated. In the present study, we obtained the expression profile of circRNAs from a GEO database (GSE96964) and identified hsa_circ_0000073 as a highly expressed candidate in OS. Overexpression of hsa_circ_0000073 accelerated the proliferation, migration, invasion and MTX resistance of OS cells, and knockdown of hsa_circ_0000073 resulted in the opposite effects. Mechanistically, hsa_circ_0000073 upregulated NRAS expression by targeting miR-145-5p and miR-151-3p in OS cells. In addition, the promotion of OS progression by hsa_circ_000007 was blocked by miR-145-5p and miR-151-3p-mediated NRAS inhibition. In conclusion, hsa_circ_0000073 facilitated the proliferation, migration, invasion and MTX resistance of OS cells through the inhibition of miR-145-5p and miR-151-3p-mediated downregulation of NRAS.

## INTRODUCTION

Osteosarcoma (OS), a highly invasive and metastatic disease, is the most universal primary malignant bone tumor; it has an incidence of approximately 4.4/100,000 [1]. With the development of adjuvant chemotherapy drugs and technologies, these treatments can be applied not only for the removal of cancer cells but also for the evaluation of surgical procedures and for the prevention of early metastasis of OS cells [2]. Statistics reveal that the 5-year survival rate of patients with nonmetastatic OS can reach 65-70%, and only 10-20% of patients require surgical intervention [3]. Despite this, the prognosis for patients with metastatic OS is not encouraging, and the 5-year survival rate for patients with pulmonary metastasis is only 20% [4]. Therefore, an in-depth understanding of the mechanism of metastasis is of great significance for improving the therapeutic effect of OS.

Circular RNAs (circRNAs), a class of covalently closed endogenous RNA molecules, have begun to be extensively studied because of their unique structure and potential function [5]. In recent years, circRNAs have been reported to serve as sponges on miRNAs, thereby affecting gene transcription or RNA-binding protein interactions [6]. Many studies have also revealed that circRNAs are specifically expressed in tumor tissues and have significant effects on cancer progression [7–9]. At present, there have been roles reported in OS for certain circRNAs, such as hsa_circ_0081001 [10], hsa_circ_0001564 [11], hsa_circ_001569 [12], hsa_circ_0002052 [13] and hsa_circ_0051079 [14]. However, research on the function and mechanism of circRNAs in osteosarcoma is still just beginning.

In the present study, we screened the expression profile of circRNAs in methotrexate (MTX)-resistant or nonresistant OS cells from a Gene Expression Omnibus (GEO) database (GSE96964). We identified a novel circRNA, hsa_circ_0000073 (hsa_circ_001069), and we explored its biological roles in the progression of OS. In addition, we also found that hsa_circ_0000073 could positively regulate NRAS expression in OS by competitively binding with miR-145-5p and miR-151-3p. Our results revealed that the hsa_circ_0000073/(miR-145-5p, miR-151-3p)/NRAS axis in OS might provide potential biomarkers and therapeutic targets for OS.

## RESULTS

### The circRNA hsa_circ_0000073 was identified in a screen because of its high expression in OS

**Figure 1:**
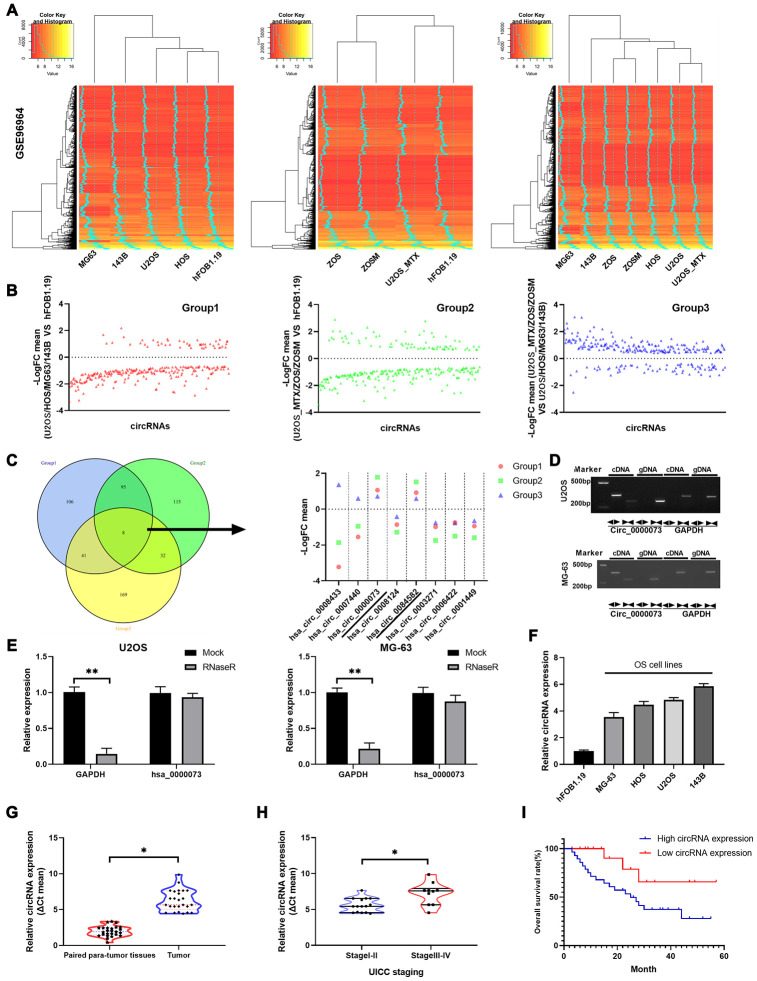
**Hsa_circ_0000073 was identified in a screen and was highly expressed in OS.** (**A**) Hierarchical clustering was applied to analyze the expression profiles of circRNAs of the GSE96964 database in OS cells (MG63, 143B, U2OS and HOS), human osteoblast cells (hFOB 1.19) and OS MTX-resistant cells (ZOS, ZOSM, and U2OS_MTX). (**B**) Levels of circRNAs were displayed in 3 groups. (**C**) Venn diagrams showed that there are 8 circRNAs among the three groups, and the levels of the 8 circRNAs are also shown. (**D**) Hsa_circ_0000073 expression was confirmed in cDNA and gDNA of U2OS and MG-63 cells using divergent primers and convergent primers. (**E**) The stability of hsa_circ_0000073 was determined in U2OS and MG-63 cells after treatment with RNase R by qRT-PCR assays. (**F**) Hsa_circ_0000073 expression was further determined using qRT-PCR analysis in hFOB 1.19 and OS cells (MG63, 143B, U2OS and HOS). (**G**) qRT-PCR analysis of hsa_circ_0000073 in OS and paired paratumor tissues. (**H**) qRT-PCR analysis of hsa_circ_0000073 in stage I-II and stage III-IV (UICC staging). (**I**) Kaplan-Meier analysis was applied to estimate the survival curve of hsa_circ_0000073 in OS. *P<0.05, **P<0.01.

We preliminarily explored the circRNA expression profile of a publicly available OS dataset. The expression profiles of circRNAs from GSE96964 were determined by hierarchical clustering of OS cells (MG63, 143B, U2OS and HOS), human osteoblast cells (hFOB 1.19) and OS MTX-resistant cells (ZOS, ZOSM, and U2OS_MTX) (Figure 1A). The fold changes were determined for circRNAs that were differentially expressed between U2OS, HOS, MG-63, 143B and hFOB 1.19 cells (Group 1), between U2OS_MTX, ZOS, ZOSM and hFOB 1.19 cells (Group 2), and between U2OS_MTX, ZOS, ZOSM and U2OS, HOS, MG63, 143B cells (Group 3) (Figure 1B). Through Venn diagrams, we discovered that 8 circRNAs were identified in all three groups. Then, the fold change levels of the 8 circRNAs were displayed: hsa_circ_0000073 and hsa_circ_0084582 were upregulated in OS or MTX-resistant OS among the three groups, while hsa_circ_0003271, hsa_circ_0006422 and hsa_circ_0001449 were downregulated (Figure 1C). Hsa_circ_0000073 (Supplementary Figure 1) was chosen for the research target because it had the most significant increase.

First, the circular form of hsa_circ_0000073 was assessed by using divergent primers and the cDNA from U2OS and MG-63 cells (Figure 1D). We also found that the expression of hsa_circ_0000073 was not different between an RNase R treatment group and a mock treatment group, suggesting the strong stability of hsa_circ_0000073 in U2OS and MG-63 cells (*P*<0.01, Figure 1E). In addition, our results showed that hsa_circ_0000073 was dramatically upregulated in OS cells and tissues (*P*<0.05, Figure 1F and 1G). Moreover, our data revealed that high expression of hsa_circ_0000073 was associated with poor stage and poor survival of OS (*P*<0.05, Figure 1H and 1I). In summary, we found that hsa_circ_0000073 was successfully identified from a screen and was highly expressed in OS.

### Hsa_circ_0000073 induced proliferation, migration and invasion of OS cells *in vitro*

**Figure 2:**
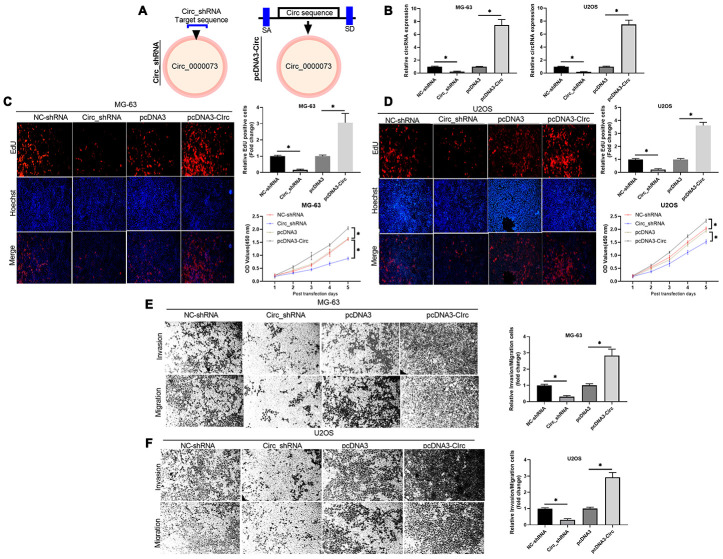
**Hsa_circ_0000073 induced proliferation, migration and invasion of OS cells *in vitro*.** (**A**) Using hsa_circ_0000073 sequence information, shRNAs and hsa_circ_0000073 overexpression plasmids of were constructed. (**B**) The overexpression and knockdown effects in MG-63 and U2OS cells were analyzed by qRT-PCR. (**C**, **D**) Edu staining and CCK-8 assays were conducted to confirm the influences of hsa_circ_0000073 overexpression and knockdown on the proliferation of MG-63 and U2OS cells. Magnification, ×100; Scale bar=100 μm. (**E**, **F**) Transwell assays were adopted to assess the changes in migration and invasion abilities in hsa_circ_0000073-overexpressing and hsa_circ_0000073-silenced MG-63 and U2OS cells. Magnification, ×100; Scale bar=100 μm. *P<0.05.

To investigate whether hsa_circ_0000073 could be involved in OS progression, two shRNAs and an overexpression plasmid of hsa_circ_0000073 were constructed (Figure 2A). Meanwhile, the transfection effects of the two shRNAs were verified in U2OS and MG-63 cells, and shRNA01 was selected for further experiments (Supplementary Figure 2A, 2B). Our results first showed that hsa_circ_0000073 was observably upregulated in the overexpression group, and hsa_circ_0000073 was significantly downregulated in the shRNA transfection group (*P*<0.05, Figure 2B). Next, the results from Edu staining revealed that knockdown of hsa_circ_0000073 notably reduced the proliferation abilities of U2OS and MG-63 cells, and overexpression of hsa_circ_0000073 resulted in a proliferation effect that was opposite to that of the hsa_circ_0000073 knockdown (*P*<0.05, Figure 2C and 2D). We also revealed that OS cell proliferation results revealed by the CCK-8 assay were similar to those of the Edu staining assay (*P*<0.05, Figure 2C and 2D). Moreover, our results certified that increased migration and invasion abilities were observed in hsa_circ_0000073-overexpressing U2OS and MG-63 cells, while sharp reductions in migration and invasion were discovered in hsa_circ_0000073-silenced U2OS and MG-63 cells (*P*<0.05, Figure 2E and 2F). Taken together, our data demonstrated that hsa_circ_0000073 can significantly promote the proliferation, migration and invasion of OS cells.

### Hsa_circ_0000073 accelerated MTX resistance and proliferation of MTX-resistant OS cells *in vitro*

**Figure 3:**
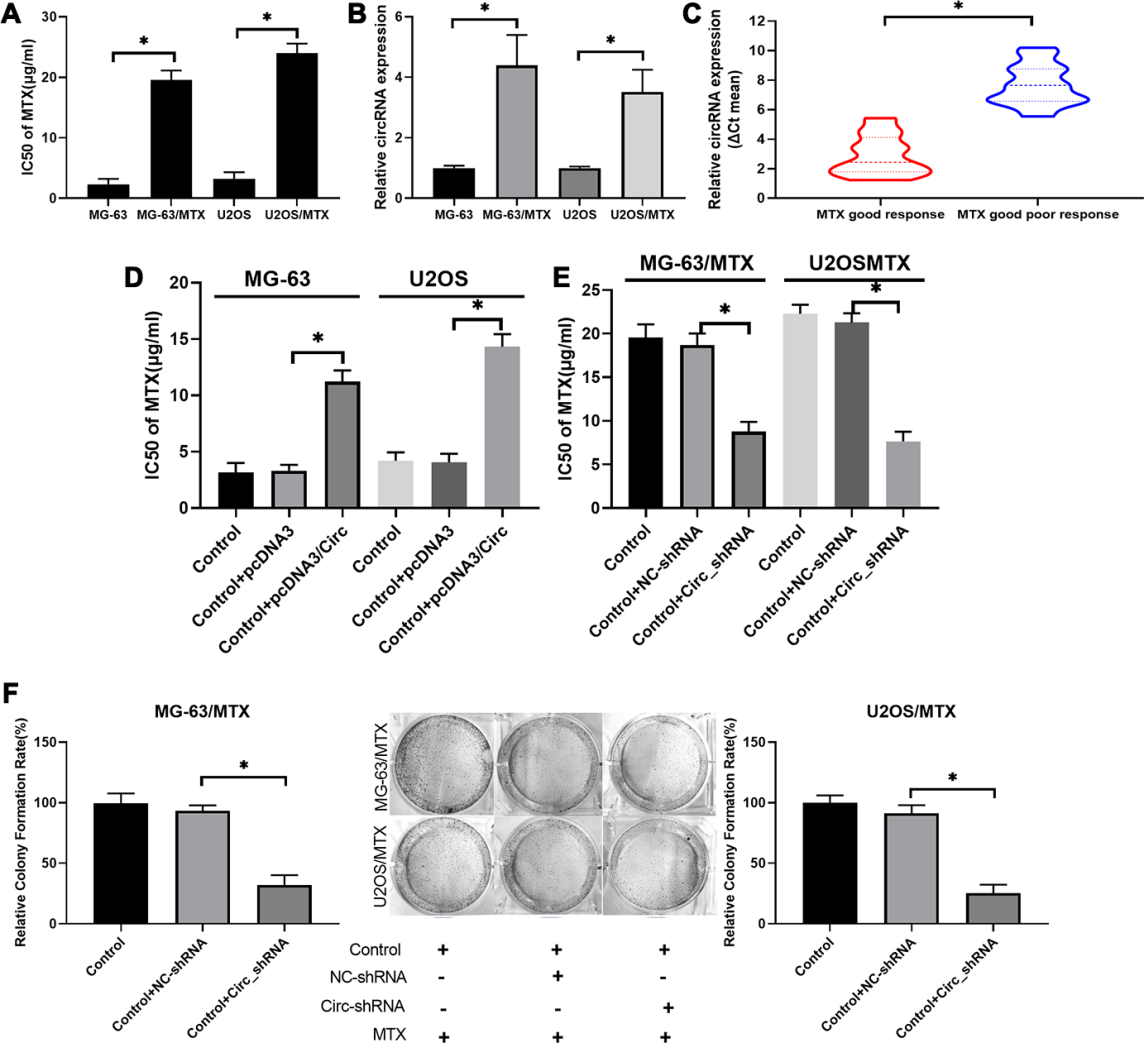
**Hsa_circ_0000073 accelerated acquisition of MTX resistance and proliferation of MTX-resistant OS cells *in vitro*.** (**A**) The IC50 of MTX was examined via CCK-8 assay in MTX-resistant MG-63 and U2OS cells. (**B**) Hsa_circ_0000073 expression was monitored through qRT-PCR assay in MTX-resistant MG-63 and U2OS cells. (**C**) Hsa_circ_0000073 expression was determined by qRT-PCR in OS patients with good or poor response to MTX. (**D**, **E**) After hsa_circ_0000073 overexpression or knockdown, the IC50 of MTX was determined using a CCK-8 assay in MG-63 and U2OS cells. (**F**) The impact of hsa_circ_0000073 knockdown on cell proliferation was assessed by colony formation assay in MTX-resistant MG-63 and U2OS cells. *P<0.05.

To investigate the impacts of hsa_circ_0000073 on the resistance and cytotoxicity of MTX-resistant OS cells, the IC50 of MTX was evaluated by CCK-8 assay. We discovered that the IC50 of MTX was dramatically increased in MG-63/MTX and U2OS/MTX cells relative to MG-63 and U2OS cells, suggesting that the inhibition rates of MTX on MG-63 and U2OS cells were significantly enhanced compared with those of MG-63/MTX and U2OS/MTX cells (*P*<0.05, Figure 3A). Meanwhile, we revealed that hsa_circ_0000073 expression was also markedly elevated in MTX-resistant MG-63 and U2OS cells with respect to MG-63 and U2OS cells (*P*<0.05, Figure 3B). Additionally, hsa_circ_0000073 expression was higher in OS patients with an inadequate response to MTX than it was in those with a good response (*P*<0.05, Figure 3C). Moreover, our results revealed that overexpression of has_circ_0000073 prominently raised the IC50 of MTX in MG-63 and U2OS cells (*P*<0.05, Figure 3D), and knockdown of hsa_circ_0000073 notably reduced the IC50 of MTX in MG-63/MTX and U2OS/MTX cells (*P*<0.05, Figure 3E). The results of the colony formation assay also showed that knockdown of hsa_circ_0000073 significantly decreased the proliferation of MG-63/MTX and U2OS/MTX cells (*P*<0.05, Figure 3F). In summary, our results showed that MG-63/MTX and U2OS/MTX cells were resistant to MTX, and hsa_circ_0000073 could further facilitate MTX resistance in MTX-resistant OS cells.

### Knockdown of NRAS notably suppressed hsa_circ_0000073-mediated proliferation, migration and invasion of OS cells

**Figure 4:**
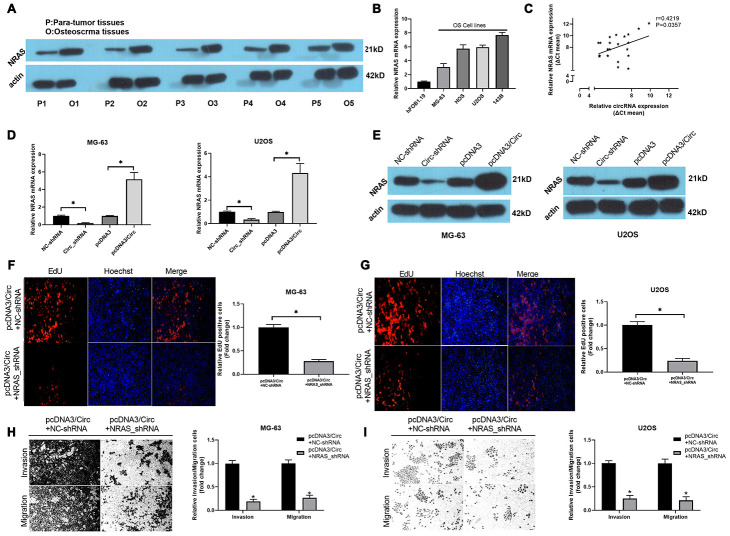
**Knockdown of NRAS notably suppressed hsa_circ_0000073-mediated proliferation, migration and invasion of OS cells.** (**A**) Western blot analysis of NRAS expression in OS and paired paratumor tissues. (**B**) NRAS expression was also evaluated by qRT-PCR analysis in hFOB 1.19 and OS cells. (**C**) The link between NRAS and hsa_circ_0000073 expression was confirmed through correlation analysis (r=0.4219, *P*=0.0357). (**D**, **E**) After hsa_circ_0000073 overexpression or knockdown in MG-63 and U2OS cells, NRAS expression was assessed by qRT-PCR and western blot assays. (**F**, **G**) MG-63 and U2OS cells were cotransfected with hsa_circ_0000073 plasmid and NRAS shRNAs. Edu staining was utilized in the transfected MG-63 and U2OS cells. Magnification, ×100; Scale bar=100 μm. (**H**, **I**) Migration and invasion capacities of the MG-63 and U2OS cells cotransfected with hsa_circ_0000073 plasmid and NRAS shRNAs were tested via Transwell assay in. Magnification, ×100; Scale bar=100 μm. *P<0.05.

Subsequently, the results of western blot analysis found that NRAS was observably upregulated in OS tissues when compared to paired para-tumor tissues (Figure 4A). Meanwhile, we revealed that NRAS was memorably increased in OS cells with respect to hFOB 1.19 cells (Figure 4B). There was a good correlation between the expression of NRAS and hsa_circ_0000073 (r=0.4219, *P*=0.0357, Figure 4C). In cell-based experiments, we first revealed that hsa_circ_0000073 knockdown significantly downregulated the level of NRAS; additionally, hsa_circ_0000073 overexpression dramatically upregulated the level of NRAS (*P*<0.05, Figure 4D and 4E). Functional experiments further showed that silencing NRAS prominently suppressed the proliferation of MG-63 and U2OS cells, which was induced by hsa_circ_0000073 overexpression (*P*<0.05, Figure 4F and 4G). Simultaneously, Transwell assays showed that silencing NRAS could also significantly reduce the migration and invasion of MG-63 and U2OS cells, which were promoted by hsa_circ_0000073 overexpression (*P*<0.05, Figure 4H and 4I). Consequently, we concluded that NRAS was closely related to the regulation of hsa_circ_0000073 on OS functions.

### The regulatory relationship among hsa_circ_0000073, miR-145-5p, miR-151-3p and NRAS in OS

**Figure 5:**
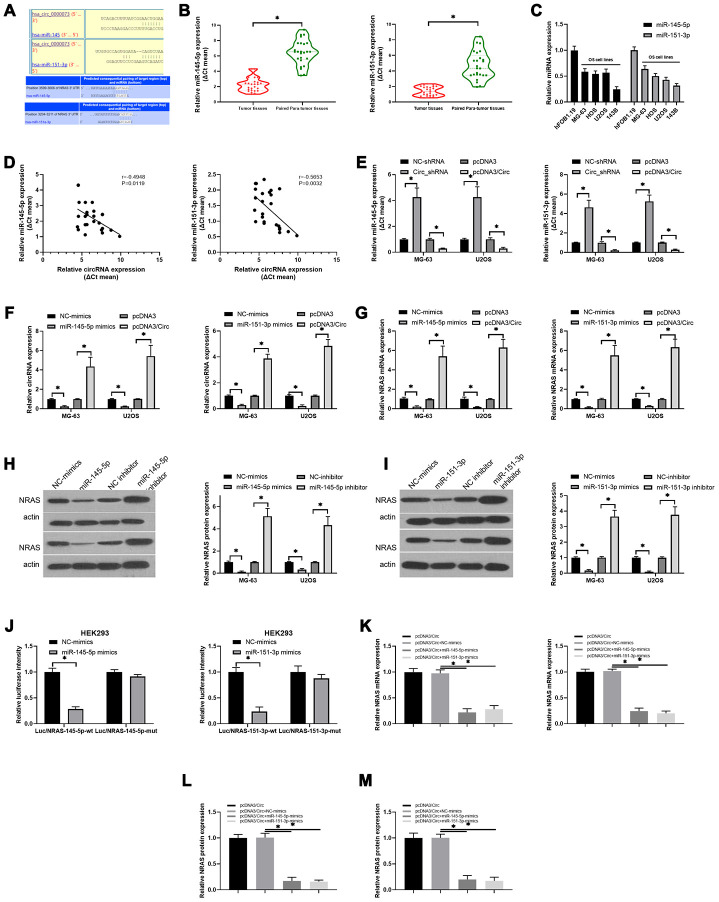
**The regulatory relationship among hsa_circ_0000073, miR-145-5p, miR-151-3p and NRAS in OS.** (**A**) The binding sites between hsa_circ_0000073 and miR-145-5p or miR-151-3p. (**B**) qRT-PCR analyses of miR-145-5p and miR-151-3p in OS and paired paratumor tissues. (**C**) MiR-145-5p and miR-151-3p expression in hFOB 1.19 and OS cells was monitored by qRT-PCR assay. (**D**) The correlativity between hsa_circ_0000073 and miR-145-5p or miR-151-3p expression in OS tissues. (**E**) qRT-PCR analyses of miR-145-5p and miR-151-3p in hsa_circ_0000073-overexpressing and hsa_circ_0000073-silenced MG-63 and U2OS cells. (**F**–**G**) MG-63 and U2OS cells were cotransfected with the hsa_circ_0000073 plasmid and miR-145-5p or miR-151-3p mimics, and the levels of hsa_circ_0000073 and NRAS were determined through qRT-PCR. (**H**, **I**) Western blot analysis of NRAS expression in MG-63 and U2OS cells after transfection with miR-145-5p or miR-151-3p mimics and inhibitors. (**J**) The luciferase intensities between NRAS and miR-145-5p or miR-151-3p were assessed via dual-luciferase reporter gene assay. (**K**–**M**) qRT-PCR and western blot assays were performed to verify the impacts of hsa_circ_0000073 and miR-145-5p or miR-151-3p on NRAS expression. *P<0.05.

Next, through analysis of the circDB database, we discovered that hsa_circ_0000073 had no protein translation function (Supplementary Figure 3A). Simultaneously, our results showed that hsa_circ_0000073 was mainly located in the cytoplasm (Supplementary Figure 3B). Through bioinformatics analysis, we also uncovered potential binding sites between hsa_circ_0000073 and miR-145-5p or miR-151-3p; there were also binding sites between miR-145-5p or miR-151-3p and NRAS (Figure 5A). Additionally, our qRT-PCR data revealed that both miR-145-5p and miR-151-3p were significantly downregulated in OS tissues compared to paired paratumor tissues (*P*<0.05, Figure 5B); simultaneously, miR-145-5p and miR-151-3p expression was also notably decreased in OS cell lines with respect to hFOB 1.19 cells (Figure 5C). The results of the correlation analysis further indicated that hsa_circ_0000073 was inversely related to miR-145-5p (r=-0.4948, *P*=0.0119), and hsa_circ_0000073 was also negatively correlated with miR-151-3p (r=-0.5653, *P*=0.0032) (Figure 5D). In MG-63 and U2OS cells, we then revealed that overexpression of hsa_circ_0000073 could result in significant upregulation of miR-145-5p and miR-151-3p expression, and knockdown of hsa_circ_0000073 could lead to their significant downregulation (*P*<0.05, Figure 5E). In addition, our results showed that miR-145-5p or miR-151-3p notably reduced hsa_circ_0000073 expression; hsa_circ_0000073 markedly elevated hsa_circ_0000073 expression (*P*<0.05, Figure 5F). Subsequently, we found that miR-145-5p or miR-151-3p significantly downregulated NRAS expression; additionally, hsa_circ_0000073 significantly upregulated NRAS expression (*P*<0.05, Figure 5G). The western blot data showed that both miR-145-5p mimics and miR-151-3p mimics could result in a decrease in NRAS expression; both miR-145-5p inhibitors and miR-151-3p inhibitors could lead to an increase in NRAS expression in MG-63 and U2OS cells (*P*<0.05, Figure 5H and 5I). HEK293 cells were cotransfected with luciferase reporters containing 3′-UTR sequences of NRAS and with miR-145-5p or miR-151-3p mimics. The results of the dual-luciferase reporter assay showed that miR-145-5p or miR-151-3p impaired the luciferase activity from the wild-type NRAS 3′-UTR (WT) reporter but not the reporter with the MUT 3′-UTR of NRAS, suggesting the targeted effects of miR-145-5p or miR-151-3p on NRAS (*P*<0.05, Figure 5J). Finally, we found that miR-145-5p or miR-151-3p markedly inhibited the expression of NRAS, which was induced by hsa_circ_0000073 overexpression in MG-63 and U2OS cells (*P*<0.05, Figure 5K–5M). In summary, our results suggested that hsa_circ_0000073 could upregulate NRAS by inhibiting miR-145-5p and miR-151-3p expression in OS cells.

### Hsa_circ_0000073 expedited proliferation, migration and invasion of OS by targeting miR-145-5p and miR-151-3p

**Figure 6:**
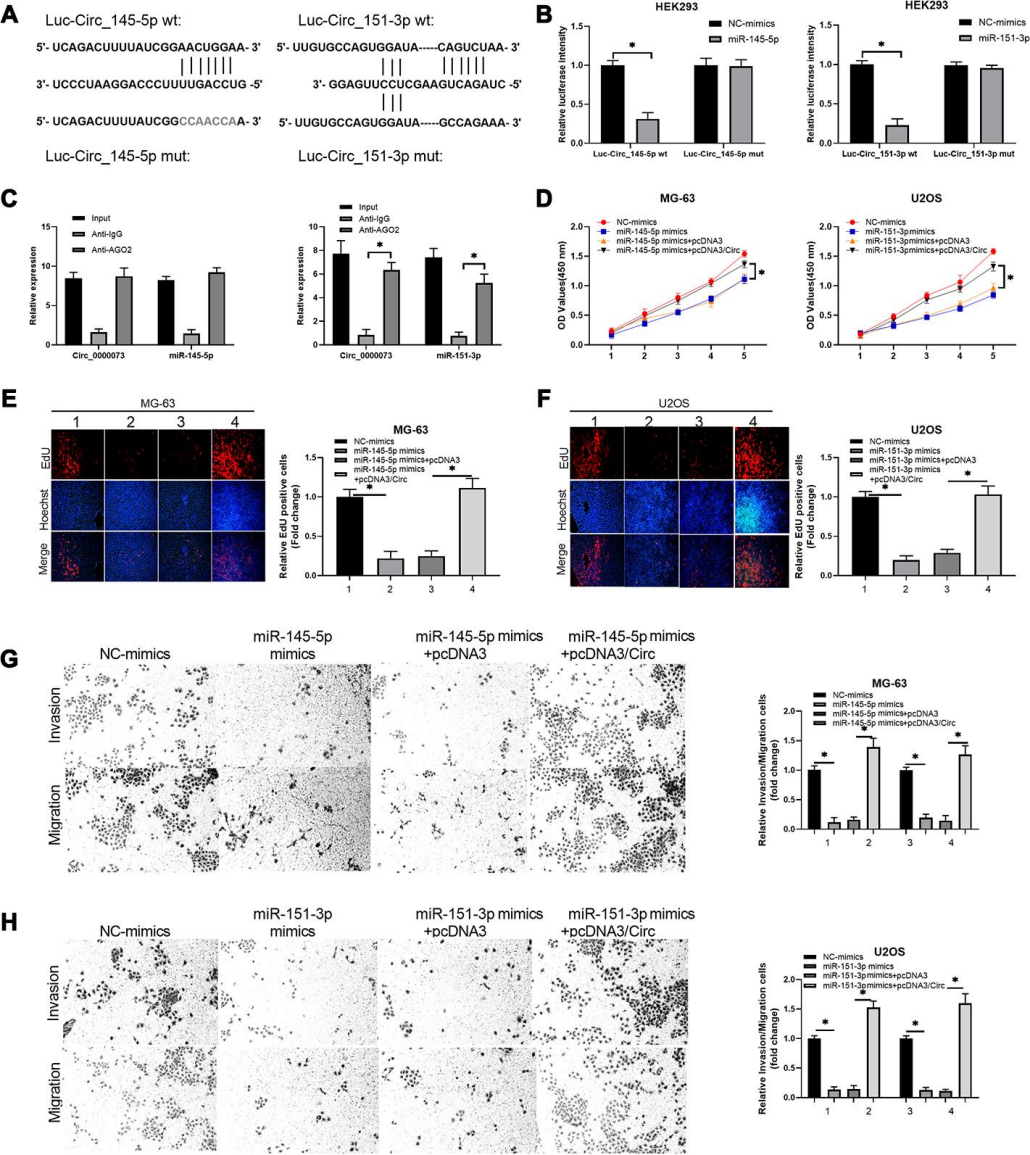
**Hsa_circ_0000073 promoted proliferation, migration and invasion of OS by targeting miR-145-5p and miR-151-3p.** (**A**) Putative miR-145-5p or miR-151-3p-binding sequence of NRAS. (**B**) Dual-luciferase reporter gene assays were applied to analyze the luciferase activity. (**C**) An AGO2 RIP assay was adopted to examine the correlations between hsa_circ_0000073 and miR-145-5p or miR-151-3p. (**D**–**F**) MG-63 and U2OS cells were cotransfected with hsa_circ_0000073 plasmid and miR-145-5p or miR-151-3p mimics. Cell proliferation was studied using CCK-8 assays and EdU staining. Magnification, ×100; Scale bar=100 μm. (**G**–**H**) Transwell assays were applied to analyze the changes in cell migration and invasion. Magnification, ×100; Scale bar=100 μm. *P<0.05.

Functionally, we further investigated whether miR-145-5p and miR-151-3p could participate in the impact of hsa_circ_0000073 on OS malignant behavior. First, we validated the direct binding between miR-145-5p or miR-151-3p and hsa_circ_0000073. We successfully constructed luciferase reporters containing hsa_circ_0000073 (Figure 6A). After cotransfection of the reporter with miR-145-5p or miR-151-3p mimics, our results showed that the mimics notably reduced the luciferase activity from the WT-hsa_circ_0000073 reporter but not the Mut-hsa_circ_0000073 reporter (*P*<0.05, Figure 6B). Meanwhile, the results of an AGO2 RIP assay showed that compared with the ani-IgG group, the expression of hsa_circ_0000073, miR-145-5p and miR-151-3p was significantly reduced in the anti-AGO2 group, suggesting direct regulation between hsa_circ_0000073 and miR-145-5p or miR-151-3p (*P*<0.05, Figure 6C). Second, our data from CCK-8 assays and EdU staining verified that miR-145-5p or miR-151-3p notably suppressed the proliferation of OS cells. This suppression could be observably attenuated by hsa_circ_0000073 overexpression (*P*<0.05, Figure 6D–6F). Next, Transwell assays revealed that miR-145-5p or miR-151-3p was effective in the inhibition of OS cell migration and invasion, while hsa_circ_0000073 overexpression reversed the inhibition mediated by miR-145-5p or miR-151-3p (*P*<0.05, Figure 6G–6H). Overall, we proved that hsa_circ_0000073 could promote malignant behavior in OS cells by regulating miR-145-5p and miR-151-3p.

### Knockdown of hsa_circ_0000073 prevented tumor growth of OS by regulating the miR-145-5p or miR-151-3p/NRAS axis *in vivo*

**Figure 7:**
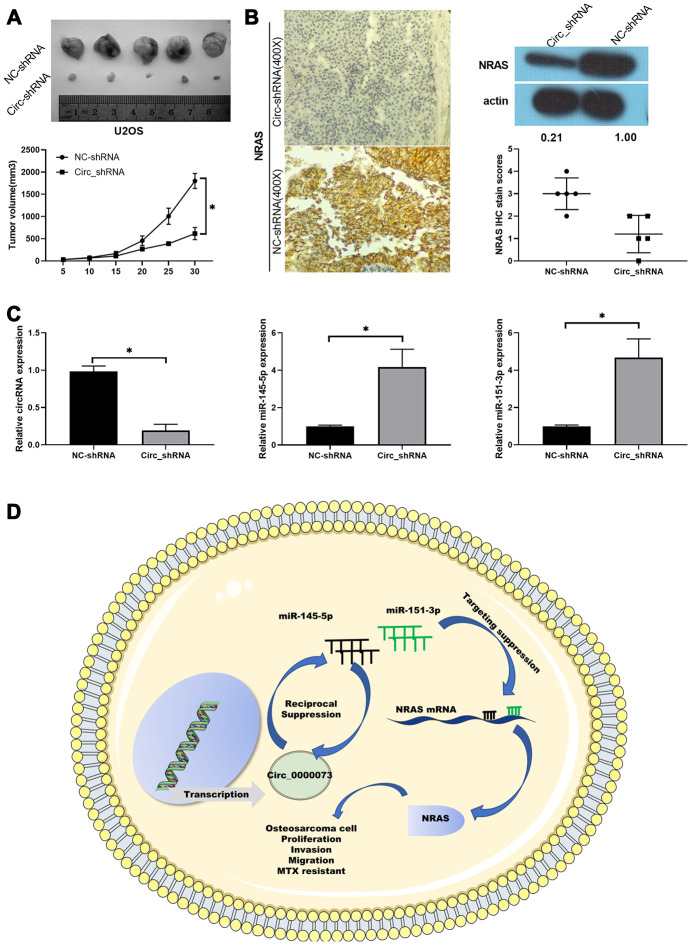
**Knockdown of hsa_circ_0000073 prevented tumor growth of OS by regulating NRAS *in vivo*.** (**A**) Collected tumors were exhibited after hsa_circ_0000073 knockdown. (**B**) After hsa_circ_0000073 knockdown, NRAS expression was assessed using IHC and western blot assays. Magnification, ×100; Scale bar=100 μm. (**C**) qRT-PCR analyses of hsa_circ_0000073, miR-145-5p and miR-151-3p. (**D**) A skeleton diagram is displayed. *P<0.05.

Furthermore, we also validated the potential significance of hsa_circ_0000073 knockdown on OS tumor growth *in vivo*, using a xenograft model. Nude mice were subcutaneously injected with MG-63 cells transfected with hsa_circ_0000073 shRNA or a NC shRNA. Throughout the growth of the tumor, we observed that the tumors grew more slowly in the hsa_circ_0000073 knockdown group than in the NC shRNA (control) group (*P*<0.05, Figure 7A). Meanwhile, the results of IHC and western blot assays revealed that knockdown of hsa_circ_0000073 markedly reduced the expression of NRAS in OS tumors (*P*<0.05, Figure 7B). In addition, qRT-PCR analysis revealed that knockdown of hsa_circ_0000073 prominently upregulated miR-145-5p and miR-151-3p in OS tumors (*P*<0.05, Figure 7C). Overall, our study proved that hsa_circ_0000073 could accelerate the proliferation, migration, invasion and MTX resistance of OS by regulating NRAS mediation by miR-145-5p or miR-151-3p (Figure 7D).

## DISCUSSION

Currently, treatment for OS is a combination of local surgery and adjuvant chemotherapy [15]. The development of adjuvant chemotherapy not only can enhance the prognosis of patients with OS but also can improve the rate of limb salvage [16]. MTX is an antitumor drug with a structure similar to folic acid, and it is widely used in a variety of tumor chemotherapies and immunosuppressive therapies [17]. Studies have revealed that MTX can dramatically improve the therapeutic effect of OS treatments [18, 19]. However, chemotherapy resistance notably affects the outcome of chemotherapy on the tumor, especially in patients with highly malignant OS. Therefore, a better understanding of the MTX resistance mechanism is of great importance for OS treatment. In our study, we used a GEO database (GSE96964), and we identified as an OS-related circular RNA hsa_circ_0000073 (hsa_circ_001069), which is located in the host gene (OMA1) and is found on chr1:58992932-59002413. We also discovered that the identified hsa_circ_0000073 was markedly upregulated in OS cells and tissues, and high expression of hsa_circ_0000073 resulted in poor OS survival. Functionally, we revealed for the first time that hsa_circ_0000073 could accelerate the proliferation, migration, invasion and MTX resistance of OS cells.

MicroRNAs, as a class of noncoding small RNA molecules, can regulate gene expression at the posttranscriptional level by binding to the 3' UTR of target mRNAs [20, 21]. Currently, it is generally believed that miRNAs are not only connected with the physiological processes of proliferation, apoptosis, proliferation, and embryonic development but can also be involved in cancer progression by acting as a carcinogen or tumor suppressor gene [21, 22]. In our study, to ascertain whether hsa_circ_0000073 participates in the ceRNA model, miR-145-5p and miR-151-3p were predicted and were shown to bind directly to hsa_circ_0000073. Meanwhile, miR-145-5p and miR-151-3p were negatively correlated with hsa_circ_0000073. Functionally, we also revealed that the inhibition of hsa_circ_0000073 on the proliferation, migration and invasion of OS cells occurred via downregulation of miR-145-5p and miR-151-3p. In previous studies, miR-145-5p and miR-151-3p have also been proven to prevent tumorigenesis [23–25]. These solid pieces of evidence support the idea that the circRNA/miRNA axis plays important roles in OS progression.

Previous studies have also shown that circRNAs can suppress mRNA expression by acting as miRNA sponges [26]. As previous studies have reported, miR-145-5p could repress the progression of bladder cancer by regulating SOX11 [27]; further, miR-145-5p could suppress the progression of laryngeal squamous cell carcinoma via FSCN1 [23]. Research has also certified that miR-151-3p could prevent the migration of breast cancer by targeting TWIST1 [25]. In our study, we found that NRAS was a direct regulatory target of miR-145-5p and miR-151-3p. NRAS could be upregulated by hsa_circ_0000073 by the inhibition of miR-145-5p and miR-151-3p in OS cells. Functionally, we also demonstrated that knockdown of NRAS also inhibited the proliferation, migration and invasion of OS cells, which were mediated by hsa_circ_0000073.

## CONCLUSION

Our study suggested that hsa_circ_0000073 could facilitate the proliferation, migration and invasion of OS cells by directing miR-145-5p or miR-151-3p regulation of NRAS. We also speculated that the hsa_circ_0000073/miR-145-5p and miR-151-3p/NRAS axes were closely connected with MTX resistance in OS. Therefore, hsa_circ_0000073, miR-145-5p, miR-151-3p and NRAS might be possible markers in OS, providing potential targets for the therapy of clinical OS patients.

## MATERIALS AND METHODS

### Clinical samples

Twenty-five pairs of OS and paired paratumor tissues were harvested at the Affiliated Huai’an Hospital of Xuzhou Medical University and the Second People’s Hospital of Huai’an. OS was confirmed in patients through pathological examination. All 25 patients received initial treatment and did not receive any antitumor therapy. We also obtained informed consent from all patients. Our research has been approved by the Ethics Committee of the Affiliated Huai’an Hospital of Xuzhou Medical University and the Second People’s Hospital of Huai’an. All tissues were extracted, labeled and immediately stored at -80 °C.

### Cell culture and treatment

Human osteoblast cells (hFOB 1.19), OS cells (MG63, 143B, U2OS, and HOS) and HEK293 cells were all obtained from ATCC (Rockville, USA). Methotrexate (MTX)-resistant cell lines (MG-63/MTX and U2OS/MTX) were formed by exposing MG63 and U2OS cells to increasing concentrations of MTX. All cells were grown in Dulbecco’s modified Eagle’s medium (DMEM, Gibco, USA) with 10% fetal bovine serum (FBS, Gibco, USA) at 37 °C in 5% CO_2_. In addition, MG-63/MTX and U2OS/MTX cells were cultured in complete medium with 1 mg/mL MTX.

### Cell transfection

Hsa_circ_0000073-overexpressing plasmid, control (pcDNA3), hsa_circ_0000073 shRNAs, NRAS-shRNAs, and negative control (NC)-shRNAs were purchased from Nanjing Dehengwen Biological Technology Co., Ltd. NC-mimics, miR-145-5p mimics, miR-151-3p mimics, NC-inhibitor, miR-145-5p inhibitor, and miR-151-3p inhibitor were purchased from GenePharma (Shanghai, China). U2OS and MG-63 cells were plated at a concentration of 1 × 10^5^ cells/well in 6-well plates and then were transfected with all plasmids, mimics and inhibitors using Lipofectamine 3000 reagent (Invitrogen) in accordance with the experimental instructions. Detailed cell phenotype assays, including CCK8, EdU stain, Transwell and colony formation assays, are shown in the supplementary methods.

### Identification of differentially expressed circRNAs

Data on circRNAs were collected from the websites of GEO (http://www.ncbi.nlm.nih.gov/geo/). GEO2R was adopted to obtain the top 250 differentially expressed circRNAs in OS cells, hFOB 1.19 and OS MTX-resistant cells.

### Agarose gel electrophoresis and qRT-PCR assays

TRIzol reagent was used to extract total RNA from OS tissues, paired paratumor tissues, and the transfected OS cells. The RNA was tested by a Nandoprop 2000 at an absorbance ratio of 260 nm to 280 nm. RNA was reverse transcribed to generate cDNA using a reverse transcription kit (Applied Biosystems; Cat. no. 4368814). PCR assays were carried out using 2% agarose gel electrophoresis to examine the levels of gene expression (cDNA and gDNA). Meanwhile, quantitative analyses of genes were determined using the SYBR Green PCR kit (Takara) based on the experimental instructions. The results of the qRT-PCR assay were standardized by 2^-ΔΔCt^. The primers used in this research are shown in Table 1.

**Table 1:** The sequences of primers in qRT-PCR assay.

**ID**	**Sequence (5’- 3’)**
GAPDH	Forward: TGTTCGTCATGGGTGTGAAC
GAPDH	Reverse: ATGGCATGGACTGTGGTCAT
U6	Forward: CTTCGGCAGCACATATAC
U6	Reverse: GAACGCTTCACGAATTTGC
Hsa_circ_0000073	Forward: AGGCCGAAGCTGACAAAAT
Hsa_circ_0000073	Reverse: CAAACCAAGGAATAGCTTCCA
NRAS	Forward: ACAGTGCCATGAGAGACCAA
NRAS	Reverse: AATCCCGTAACTCTTGGCCA
MiR-145-5p	Forward: CAGGAATCCCTTAGATGCTA
MiR-145-5p	Reverse: CCATGACCTCAAGAACAGT
MiR-151-3p	Forward: GGATGCTAGACTGAAGCTCCT
MiR-151-3p	Reverse: CAGTGCGTGTCGTGGAGT

### Western blot analysis

Total protein was extracted using RIPA lysis buffer (Beyotime; Cat. no. P0013B) with protease inhibitor, and the concentration was determined using a BCA method (Cwbio, Beijing, China; Cat. no. CW0014). Forty micrograms of protein from each group was separated using 10% SDS-PAGE (Solarbio, China), and the proteins were transferred to PVDF membranes (Millipore; cat. no. ISEQ00010). After blocking the membranes for 2 hr, primary antibodies were incubated with the membranes overnight at 4 °C. After treatment with a secondary antibody (Abcam) for 2 hr, an ECL substrate kit (Thermo Scientific) was utilized to examine the results.

### Dual-luciferase reporter assay

Wild type (WT)-pmirGLO-Hsa_circ_0000073, mutant (Mut)-pmirGLO-Hsa_circ_0000073, WT-pmirGLO-NRAS and Mut-pmirGLO-NRAS were purchased from Nanjing Dehengwen Biological Technology Co., Ltd (Nanjing, China). HEK293T cells were cotransfected with the WT or Mut plasmids and either the NC-mimics, miR-145-5p mimics or miR-151-3p mimics through the application of Lipofectamine 3000 reagent (Invitrogen). After 48 hr, a Dual-Luciferase Reporter Assay System (Promega) was used to determine the fluorescence intensity.

### Ago2-RNA immunoprecipitation (RIP) assay

RIP assays were carried out according to the manufacturer’s instructions using a Magna RIP™ RNA-binding protein immunoprecipitation kit (Millipore); HEK293 cells were used after transfection with miR-145-5p mimics, miR-151-3p mimics or NC-mimics for 48 hr. Briefly, cells were lysed and incubated with RIP buffer including magnetic beads and anti-Argonaute2 (AGO2) antibody (Millipore) or IgG (Millipore). After incubation with Proteinase K, the immunoprecipitated RNA was extracted. The results were analyzed by qRT-PCR assay.

### Statistical analysis

All measurement data are displayed as the mean ± SD. The experimental data were statistically analyzed using 20.0 SPSS software (PSS, Inc., Chicago, USA). The results were calculated via *t*-test or one-way analysis of variance. *P* <0.05 indicated that the results were statistically significant.

## Supplementary Material

Supplementary Methods

Supplementary Figures
