# Supplementary material for: Circular RNA hsa_circ_0000073 contributes to osteosarcoma cell proliferation, migration, invasion and methotrexate resistance by sponging miR-145-5p and miR-151-3p and upregulating NRAS
Source: Aging (Albany NY). 2020 Jul 24;12(14):14157–73. doi: 10.18632/aging.103423 (PMC7425447; doi:10.18632/aging.103423)
Supplement: Supplementary Methods [file aging-12-103423-s001..pdf]

SUPPLEMENTARY METHODS

CCK-8 assay

The transfected MG63, U2OS, MG-63/MTX and U2OS/MTX cells (2×10^3 cells/well) were inoculated to 96-well plates according to the grouping method. After 1, 2, 3, 4 and 5 days of incubation at 37 °C, 10 µl CCK8 solution (Beyotime, Haimen, China) was placed in each well, respectively. After reaction at 37 °C for 3 hrs, the OD value was determined at 450 nm using a microplate reader (BIOTEK, Vermont, USA). Meanwhile, the IC50 of MTX was calculated.

Edu staining

The treated OS cells (1×10^4 cells/well) during logarithmic growth were inoculated into 24-well plates and cultured overnight at 37 °C. Cells were added with 500 µL EdU solution (50 µM; Thermo Fisher Scientific) for 2 hrs at 37 °C. After washing, cells were fixed using 500 µL 4% paraformaldehyde solution (Sigma Aldrich; cat. no. 158127-100G) for 30 mins, and treated with 500 µL 2 mg/mL glycine for 5 mins. After washing, the cells were treated with 500 µL 0.5% TritonX-100 (Sigma-Aldrich; cat. no. T8787) for 10 mins. After treatment with 1 × Apollo staining solution for 30 mins in dark, cells were processed with the penetrating agent and washed using methanol and PBS, respectively. Finally, the DNA staining was performed using 1×Hoechst 33342 (Sigma). The results were obtained using a fluorescence microscopy.

Transwell assay

Cell invasion and migration were monitored using Transwell chambers (BD Pharmingen) with a pore diameter of 8 µmol/L. For cell migration, the U2OS and MG-63 cells in each group were suspended using serum-free medium. The suspended cells (200 µL, 2×10^5 cells/well) were administered to the upper of the Transwell chamber, the complete medium (600 µL) was placed into the lower chamber. After 24 hrs of culture, cells were fixed with 4% paraformaldehyde (Merck-millipore) and dyed with 4% crystal violet (Solarbio, China). The migrated cells were confirmed using a microscope. Before the cell invasion experiment, the cells were humidified with Matrigel, and the other steps were the same as the migration experiment.

Colony formation assay

The transfected U2OS and MG-63 cells (300 cells/well) were seeded into 6-well plates and cultured for 10 days under standard conditions. Cells were washed in PBS, fixed in 4% paraformaldehyde (Merck-millipore) for 15 mins and dyed with 5% crystal violet (Solarbio, China) for 3 mins.
