# Supplementary material for: Circular RNA hsa_circ_0000073 contributes to osteosarcoma cell proliferation, migration, invasion and methotrexate resistance by sponging miR-145-5p and miR-151-3p and upregulating NRAS
Source: Aging (Albany NY). 2020 Jul 24;12(14):14157–73. doi: 10.18632/aging.103423 (PMC7425447; doi:10.18632/aging.103423)
Supplement: Supplementary Figures [file aging-12-103423-s002..pdf]

SUPPLEMENTARY FIGURES

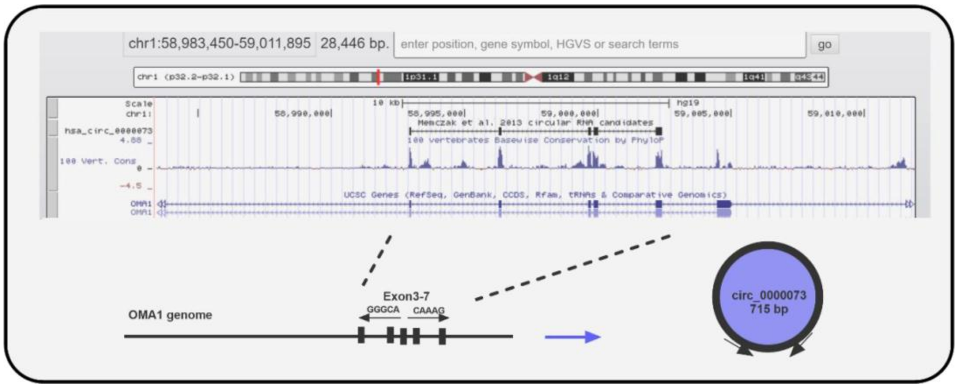

Supplementary Figure 1. The scheme of hsa_circ_0000073.

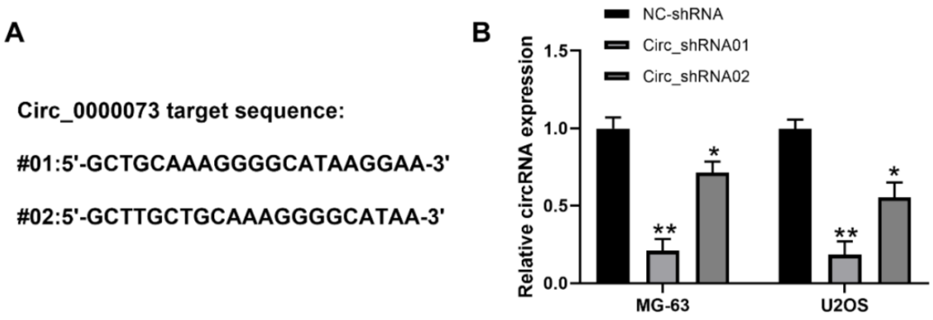

Supplementary Figure 2. (**A**) Circ_0000073 target sequence for shRNA design. (**B**) The silenced efficiencies of circ_0000073 knock down plasmids were qualified by a qRT-PCR assays, and Circ_shRNA-01 was selected in the following RNAi experiments.

A

Transcript id	Exon information			
NM_145243	**Spliced_len**	**Exon Number**	**Exon Sizes**	**Exon Offsets**
	715	5	75, 129, 108, 174, 229	0, 3340, 6692, 6897, 9252
**Protein coding potential**				
**IRES Elements**	Parameter Index			
	**Position (start--end)**	**R Score**	**With Pseudoknot (Y/N)**	
	273--409	1.583801	Y	
	636--712	1.485804	Y	
**Open Reading Frame (ORF)**	**Start Position**	**End Position**	**Protein Length**	
	233	1r+36	172 aa	
	MEEFKNDMLT EKDARYLAVK EVLCHLIECN KDVPGISQIN WVIHVVDSPI INAFVLPNGQ MFVFTGFLNS VTDIHQLSFL LGHEIAHAVL GHAAEKAGMV HLLDFLGMIF LTMIWAICPR DSLALLCQWI QSKLQEYMFN RPYSRKLEAE ADKIGLLLAA KGHKEMVAGT SS*			
	Note: (1). nr represents n rounds(n<3); (2). * represents a stop codon.			
**Protein Features**	The possibility of encoding protein is relatively low(R<1.6 or it has no open reading frame), so no protein features was predicted!			

B

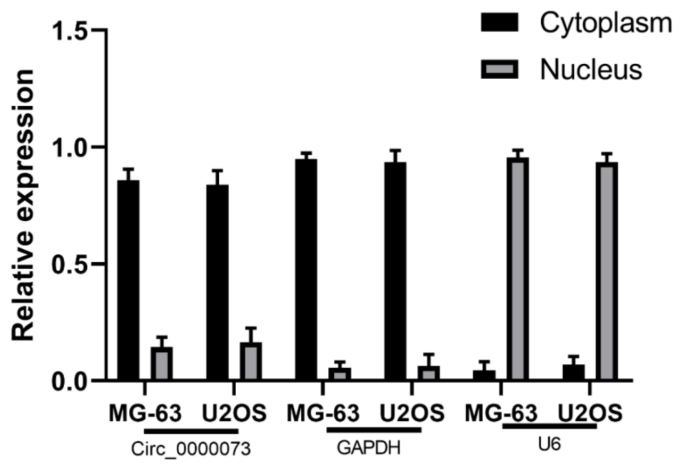

Supplementary Figure 3. (**A**) CircDB database showed that there was no translation function in hsa_circ_0000073. (**B**) The expression of circ_0000073 in U2OS cells by in-situ hybridization assay.
